# Three-dimensional evaluation of facial asymmetry and its association to occlusal and muscular imbalance in young women

**DOI:** 10.4317/jced.61985

**Published:** 2024-09-01

**Authors:** Lilian Mendes Andrade, Anna Luísa Alves Fernandes, Laís Valencise Magri, Jardel Francisco Mazzi-Chaves, Paulo Batista de Vasconcelos, Selma Siéssere, Isabela Hallak Regalo, Simone Regalo, Marcelo Palinkas

**Affiliations:** 1 DDS, PhD, Professor. Department of Basic and Oral Biology, Ribeirão Preto School of Dentistry, University of São Paulo, Brazil; 2 DDS. Department of Basic and Oral Biology, Ribeirão Preto School of Dentistry, University of São Paulo, Brazil; 3 DDS, PhD, Professor. Department of Restorative Dentistry, Ribeirão Preto School of Dentistry, University of São Paulo, São Paulo, Brazil; 4 DDS, PhD, Professor. Department of Basic and Oral Biology, Ribeirão Preto School of Dentistry and National Institute and Technology - Translational Medicine (INCT.TM), University of São Paulo, Brazil

## Abstract

**Background:**

Diagnosing asymmetries and restoring functional balance are challenges in facial rehabilitation and aesthetic procedures. This cross-sectional observational study aimed to evaluate whether occlusal imbalance and the electromyographic activity of the masseter and temporalis muscles in young women may be associated with facial asymmetry.

**Material and Methods:**

Fifty women (mean age ± standard deviation: 22.5 ± 2.7), without temporomandibular dysfunction and with balanced facial profiles, were divided into two groups categorized by receiver operating characteristic analysis: symmetric (n=25) and asymmetric (n=25). The variables included the evaluation of asymmetry through clinical examination, quantification of asymmetry using stereophotogrammetry through the root mean square method, asymmetry of occlusal contacts, and electromyographic activity of the masseter and temporal muscles in both latero-lateral and antero-posterior directions. The mean asymmetry indices were compared using Welch’s t-test and the Mann-Whitney test. The impact of occlusal and muscular imbalance on facial asymmetry was assessed through linear regression analysis.

**Results:**

A significant difference was observed between the groups in the asymmetry of occlusal contacts, with a considerable effect size (*p*<0.01 - Cohen’s d=0.73). The imbalance in the electromyographic activities of the masseter and temporal muscles was considered a predictor of facial asymmetry (F=4.00, *p*<0.02, R²=0.15).

**Conclusions:**

Occlusal imbalance and electromyographic activity of the masseter and temporal muscles are associated with facial asymmetry.

** Key words:**Facial asymmetry, masticatory muscles, stereophotogrammetry, occlusion, electromyography.

## Introduction

The increasing demand for aesthetic procedures has sparked discussions about facial symmetry and beauty standards, with some authors suggesting that facial symmetry influences facial attractiveness (1,2), while other studies indicate that proper facial proportions within normative standards also contribute to this attractiveness (3). Regardless of the aesthetic approach, facial asymmetries can indicate functional imbalance and should be assessed in the diagnosis and planning of all facial interventions.

The term “asymmetry” refers to the inequality between homologous parts and concerns the extent to which one half of an object, image, or organism differs from the other half (4). The human face exhibits bilateral symmetry, meaning it can be divided into two halves, each being the mirrored image of the other. This symmetry is approximate, as normal populations display minor random variations (5,6).

Facial asymmetries can be congenital, occurring during the formation of facial structures, or result from complex interactions during facial growth and development. (7). The etiology includes genetic influences, accidents, injuries, surgical and aesthetic interventions, aging, and dental issues, with various factors often collectively contributing to the manifestation of facial asymmetry traits (7,8).

Skeletal striated musculature is an important dynamic anatomical structure for maintaining the balance of facial structures. Uncoordinated muscle movements can alter bone homeostasis and contribute to the development of asymmetries (9). During contraction, muscles tension bones via their insertions, transmitting loads along these muscular attachments. Excessive or decreased tension in these loads can induce or reduce bone formation and resorption, thereby modifying morphology (10,11). Depending on the intensity and duration of the stimulus, these disturbances can result in facial asymmetry (12). Additionally, the distribution of occlusal contacts has been considered in relation to muscular response, suggesting that muscular balance may be associated with occlusal balance (13).

Considering the importance of muscular behavior in maintaining facial structure balance, this study aimed to assess whether occlusal imbalance and electromyographic activity of the masseter and temporal muscles are associated with facial asymmetry. The null hypothesis of this study was that facial asymmetry in young women is not associated with morphofunctional disharmony of the masticatory muscles.

## Material and Methods

-Study design and sample selection

This cross-sectional observational study was approved by the ethics committee (protocol # 59833522.0.0000.5419), and all participants were informed about the objectives and procedures. They digitally signed the informed consent form. The study was outlined in the Research Electronic Data Capture (REDCap) system (14).

The sample size was determined based on a medium effect size (d=0.4) from a study on automated facial asymmetry assessment (15). Sample size calculation was performed using G* Power 3.1.9.2 software (Franz Faul, Kiel University, Germany) with a linear regression protocol, considering a medium effect size (f=0.25), alpha error of 0.05, and beta of 0.80. The software calculated a total of 46 individuals required to comprise the sample.

Participants were recruited through an open invitation sent via email. The document included explanations about the study and provided a link to REDCap, where participants read the informed consent form. Upon acceptance, they accessed a self-report questionnaire to provide demographic and baseline data.

Participants’ questionnaire responses enabled preliminary selection, after which they were scheduled for an in-person assessment. During the onsite visit, participants underwent verification of inclusion and exclusion criteria and data collection for study variables. This process was conducted by two researchers and lasted approximately one hour. Data collection included initial clinical examination, digital occlusal analysis, electromyographic and photographic analysis, all following the same methodological sequence.

Fifty young adult women participated, aged between 18 and 30 years (mean age ± standard deviation: 22.5 ± 2.7) with a mean body mass index ± standard deviation of 22.6 ± 3.98. The participants were divided into two groups: symmetric (n=25) and asymmetric (n=25).

Inclusion criteria included a normal body mass index, a balanced facial profile, the absence of skeletal discrepancies, and a complete set of natural teeth. Exclusion criteria encompassed symptoms of temporomandibular dysfunction (assessed using the DC/TMD Symptom Questionnaire), prior facial harmonization procedures, use of anti-inflammatory or muscle relaxant medications, the presence of facial ulcers, cutaneous hypersensitivity, mandibular torus, anterior or posterior crossbite, and any environmental or functional conditions that could interfere with facial muscle evaluation.

The study variables included assessment of asymmetry through clinical examination, quantification of asymmetry using stereophotogrammetry, asymmetry of occlusal contacts, and electromyographic activity of the masseter and temporal muscles in the latero-lateral and anteroposterior directions.

-Evaluation of facial asymmetry

The variable of facial asymmetry was obtained through clinical examination conducted by two previously trained and calibrated professionals (intraclass correlation coefficient of 0.87). This assessment followed protocols described in the literature (4,7). The classification was recorded in an electronic spreadsheet as a dichotomous variable: symmetric and asymmetric.

-Stereophotogrammetry for quantification of asymmetry

The quantification of asymmetry using stereophotogrammetry was performed in the Vectra Analysis Module software using images obtained from the Vectra M3 3D imaging system (Canfield Scientific, Inc., Parsippany, NJ, EUA) (16). The images obtained were analyzed by a single trained professional. Precise delineation of the face was performed to ensure method repeatability and reliable quantification of asymmetry. Landmarks such as thrichium, frontotemporale, tragus, gonion, and menton, previously marked on the face with a hypoallergenic delineating pen, were used to delineate the area of interest.

Using the area delineation tool, a sequence of points was marked approximately 1 cm apart, starting from the thrichium and following the hairline insertion line on the right side of the monitor, up to the frontotemporale alignment. From this point, the image was rotated to view the tragus.

Continuing along the hairline and passing over the tragus, the point marking proceeded in a straight line to the gonion. The image was slightly rotated upwards to expose the edge of the mandible body down to the menton, where points continued to be marked, following the bony contour of the mandibular edge.

The area delineation process continued to the left side, following the same described points until reaching the thrichium again. All images outside the delineated area were digitally removed, leaving only the face without ears, neck, and hair, representing the analysis area.

The face image was manually aligned and centered within a grid space, referencing the x, y, and z planes. A symmetry plane was automatically calculated, ensuring the most appropriate symmetry axis. Each image was then copied and mirrored along the x-plane, creating another image with the right and left sides inverted.

The original and mirrored images were overlaid, and an algorithm automatically calculated the distance between corresponding points on the two images. The measurement between the original facial surface and its mirrored image was automatically performed, computing the root mean square of the average distances. The root mean square values were used as variables to quantify facial asymmetry and categorize participants into two groups: symmetric and asymmetric.

-Analysis of Occlusal Contact Asymmetry

The assessment of occlusal contact asymmetry was performed using the T-SCAN® system (Tekscan Inc., Arbor MI, USA) (17,18). A piezoelectric pressure sensor, attached to a handpiece, was placed in the mouth like a dental impression tray. The participant was instructed to bite down with maximum voluntary contraction. The Tekscan® software automatically calculated the intensity of occlusal contacts, considering 100% as the maximum point of contact distribution. The percentage of contacts on the right and left sides was recorded in an electronic spreadsheet, and the occlusal contact asymmetry index was automatically calculated. The formula for calculating the asymmetry index is as follows: (% right side) – (% left side) / (% right side) + (% left side) * 100 (19).

-Asymmetry in the electromyographic activity of the masseter and temporal muscles

The electromyographic activities of the masseter and temporal muscles were recorded using the BTS-TMJOINT electromyograph (BTS Medical, Garbagnate Milanese, Italy) (20,21). The participant was positioned in a comforTable chair without a headrest, maintaining a natural head posture, feet flat on the floor, and hands resting on the legs. The skin was cleaned with 70% ethyl alcohol (Cooperalcool, Piracicaba, São Paulo, Brazil) in the area where electrodes were placed to reduce skin impedance by removing residues and oils (22). The participant was instructed to clench their teeth so that the masseter muscles could be palpated and located.

The electrodes were positioned parallel to the fibers of the masseter and temporal muscles, following the recommendations of SENIAM (Surface EMG Non-Invasive Assessment of Muscles) (23). The pre-determined protocols from the BTS Dental Contact Analyzer software were used, with normalization of the electromyographic signal performed using cotton rolls, followed by recording the activity with maximum voluntary contraction of the teeth in contact.

The software automatically calculated asymmetry indices defined by the developer as asymmetries in electromyographic activities of the masseter and temporal muscles in the latero-lateral and anteroposterior directions (19). The asymmetry index in the electromyographic activity of the masseter and temporal muscles in the latero-lateral direction compared the activity of these muscles between the left and right sides, indicating asymmetry in muscle pairs in the lateral direction. The asymmetry index in the electromyographic activity of the masseter and temporal muscles in the anteroposterior direction evaluated anteroposterior asymmetry, estimating the prevalence of the right and left anterior temporalis muscle pairs relative to the right and left masseter muscle pairs. The data were transferred to an electronic spreadsheet, considering the absolute value of all indices.

-Statistical Analysis

All data analysis procedures were conducted using jamovi 2.3.17 software (the jamovi project, retrieved from https://www.jamovi.org). The operator’s repeatability in obtaining the facial asymmetry index using root mean square in stereophotogrammetry was verified through the intraclass correlation coefficient, measured across images of 10 participants with a 15-day interval between analyses.

The categorization of symmetric and asymmetric groups was conducted through Receiver Operating Characteristic (ROC) curve analysis (24). The ROC curve analysis compared the gold standard classification of facial asymmetry obtained from clinical examination to the root mean square scores of the participants. It identified the point where these two assessments coincided the most, which was considered the cutoff point for defining the groups.

The normality of the data and homogeneity of variances were tested using the Shapiro-Wilk and Levene’s tests, respectively. The data for occlusal contact asymmetry and asymmetries in electromyographic activities of the masseter and temporal muscles in both latero-lateral and anteroposterior directions did not exhibit normal distribution (*p*<0.05). Additionally, the data for occlusal contact asymmetry did not show homogeneity of variances (*p*<0.05). Therefore, differences between groups in the variables of asymmetries in electromyographic activities of the masseter and temporal muscles in latero-lateral and anteroposterior directions were evaluated using the Mann-Whitney U test. For the variable of occlusal contact asymmetry, groups were compared using Welch’s t-test.

 The asymmetry indices in the electromyographic activities of the masseter and temporal muscles, in both latero-lateral and anteroposterior directions, were included as predictors of facial asymmetry in the linear regression model, with occlusal contact asymmetry as a controlled variable.

## Results

An intra-class correlation coefficient of 0.945 demonstrated excellent agreement between the root mean square measurements taken 15 days apart, suggesting the reliability of stereophotogrammetry for quantifying asymmetry.

A ROC analysis produced a statistically significant curve (AUC 0.900). The result indicated that asymmetric participants had a higher root mean square in 90% of cases compared to symmetric participants. Participants with a root mean square up to 0.68 were categorized as the symmetric group, while those with a root mean square above 0.68 were allocated to the asymmetric group.  Table 1 presents the description of the evaluated groups. No significant differences were found between the groups for asymmetries in electromyographic activities of the masseter and temporalis in both latero-lateral and anteroposterior directions ( Table 2). However, significant differences were observed between the groups in occlusal contact asymmetry, with a considerable effect size (*p*<0.01, Cohen’s d = 0.73) ( Table 3). The asymmetric group consistently showed higher values than the symmetric group for the variable of asymmetries in electromyographic activities of the masseter and temporal in the latero-lateral direction. The results of the linear regression revealed that the asymmetries in electromyographic activities of the masseter and temporalis n the latero-lateral direction influenced facial asymmetry (F=4.00, *p*<0.02, R²=0.15). However, the asymmetry index of electromyographic activity of the masseter and temporal muscles in the anteroposterior direction did not impact the prediction of facial asymmetry ( Table 4).

## Discussion

The null hypothesis of this study was rejected, as it was found that facial asymmetries in young women are associated with morphofunctional disharmony of the masticatory musculature. These asymmetries may be directly linked to altered functional activity, whose is multifactorial and may progress due to frequent asymmetric muscle stimuli.

This study highlighted facial asymmetry as a potential outcome. It underscored the extent to which functional imbalance could impact the harmony of the human face. Facial asymmetry, in its three-dimensional expression, was evaluated using stereophotogrammetry technique. The diversity of methods and protocols used in previous studies have made it challenging to compare results (25-27).

Some authors have analyzed asymmetries in facial thirds yielding contrasting results. The lower third of the face has sometimes been considered the most asymmetric (28,29), while others authors have suggested greater asymmetry in the middle and upper thirds. Likewise, studies disagree on which side, right or left, is predominantly larger (30,31).

In this study, an analysis of the entire face was conducted without dividing it into thirds, considering that the insertions of the masseter and temporal muscles span the upper, middle, and lower facial thirds. Therefore, the morphological effects associated with functional imbalance of these muscles would not be limited to a specific facial third but could reflect across the entire face.

Unlike previous studies, the aim of root mean square quantification was not to establish a diagnostic reference for facial asymmetries (15,32). This study utilized root mean square as a starting point to define scores for a specific sample, composed of young, healthy women with a good maxillo-mandibular relationship. A statistically defined cutoff point was used to classify participants as symmetrical and asymmetrical so that their facial imbalance scores could be compared with occlusal and muscular asymmetry scores.

The literature offers various analyses of root mean square for identifying facial asymmetries (33). Authors have investigated asymmetries across different facial patterns, all candidates for orthognathic surgery, and found mean values of 2.85 (sd = 1.54) (32). Another study aimed to established normative data for facial asymmetry, defining RMS above 1.0 as visibly asymmetric (34). However, the lower values found in the current study are justified because a homogeneous sample of participants with a normofacial profile was selected, in contrast to findings in prior research

The method of obtaining root mean square through stereophotogrammetry presented in this study demonstrated excellent reproducibility. The good results were attributed to the prior marking of facial landmarks and the detailed protocol for image cropping. This approach ensured that the images exhibited the same facial exposure pattern, delineating the area of interest with maximum accuracy. Consequently, the baseline and mirrored images showed similar dimensions, ensuring the quality of overlays and measurements.

The choice of a homogeneous sample with mild asymmetries controlled for confounding factors associated with dentofacial changes. From the selected normal profiles, characterized as Class I and mesofacial, a good functional pattern was expected (35). Therefore, the small morphological changes could be more easily associated with functional alterations.

The results detected, with a considerable effect size, that asymmetrical individuals exhibited greater asymmetry in occlusal contacts (36).

It is known that occlusal changes directly affect the pattern of muscle contraction, and significant alterations in electromyographic activity of the temporal muscle have been demonstrated in the simulation of interferences during various mandibular movements (13). This muscle acts as a jaw positioner to achieve proper occlusal contact during closure. Prolonged contraction of the temporal muscles in the presence of interference promotes tension in the muscle region, leading to uncoordinated hyperactivity (37). The temporal muscle originates along the temporal line of the parietal and frontal bones and runs along the zygomatic arch until it inserts onto the coronoid process. In other words, it extends from the neurocranium to the viscerocranium. Some authors have demonstrated, through finite element analysis, how deformation occurs in these regions during chewing, while other studies have identified the association between cranial morphology and the cross-sectional area of the temporal muscle (38,39). When considering that facial asymmetry may be associated with underlying abnormalities in bone structure, it is necessary to understand whether uncoordinated muscular tensions can lead to bone modifications over time and result in visible facial asymmetries.

In the linear regression model, uncoordinated electromyographic activity, measured by the asymmetry index in the electromyographic activity of the masseter and temporal muscles in the lateral direction and controlled by the variable asymmetry of occlusal contacts, indicated that approximately 15% of the variation in facial asymmetry could be explained by muscular asymmetry. This result does not imply causality, as facial asymmetry can be explained by many other factors, but it suggests that muscular issues should be considered relevant in daily clinical practice.

Here, it is pertinent to discuss what is considered normal in the context of continuous stimuli, such as masticatory function. If normalcy implies the absence of symptoms (40), we can expect the stomatognathic system to achieve functional balance. However, this might not be possible without bone and muscle rearrangement, which could likely result in visible facial asymmetry, albeit subtle.

These minor asymmetries are a routine aspect for professionals in their daily practice, and to date, there are no longitudinal studies that track the evolution of functional adaptation in this context. It may be necessary not only to standardize reference values but also to categorize patterns of functional response to deviations from morphological normality. This approach would provide a clearer criterion for determining what should be corrected versus preserved.

This study had some limitations, such as the lack of information on participants’ dominant side, preferred chewing side, and midline deviation, factors that could have influenced the results if included in the multivariate analysis. Assessment of bone asymmetries would also have added relevant data to the study. The absence of bone morphology analyses was justified due to ethical concerns regarding the use of ionizing radiation. Using a sample with prior imaging exams could contribute to these analyses.

Despite these limitations, a replicable methodology was presented for quantifying facial asymmetry. Based on the analyses conducted, it is suggested to conduct further studies using similar methodologies, including individuals with different facial patterns, along with longitudinal monitoring of morphological and functional parameters. Analysis of other conditions, such as dysphagia and respiratory disorders, would also be relevant, considering the complexity and diversity of functions of the stomatognathic system.

Moreover, facial asymmetries involve multidisciplinary discussions, both in terms of diagnosis and proposed treatment. It is hoped that understanding occlusal and muscular associations with facial asymmetry will help identify undiagnosed functional imbalances, which could lead to incorrect procedures, undesired outcomes, and relapses in various interventions performed on the human face.

## Conclusions

The authors of this study suggest that occlusal imbalance and disharmony in electromyographic activity of the masseter and temporal muscles in young women are associated with facial asymmetry. Therefore, these factors should be considered in the planning of aesthetic and functional rehabilitative treatments involving the human face.

## Figures and Tables

**Table 1:** Description of the groups classified based on ROC analysis.

Groups	N	Age	Body mass index
Symmetric	25	22.5 ± 4.1	21.33 ± 4.04
Asymmetric	25	23.6 ± 4.1	22.33 ± 1.53

**Table 2:** Difference between symmetric and asymmetric groups for ASYM e ATIV.

Variables	Estatística U	p-value	Average difference	Effect Size
ASYM	244	0.184	1.100	0.2208
ATIV	293	0.705	-0.590	0.0640

ASYM. asymmetry in the electromyographic activity of the masseter and temporal muscles in the latero-lateral direction; ATIV. asymmetry in the electromyographic activity of the masseter and temporal muscles in the anteroposterior direction. Shapiro Wilk normality test and Mann Whitney test (*p*<0.05).

**Table 3:** Difference between symmetric and asymmetric groups for ACO.

Variable	Statistic (t- Welch)	gl	p-value	Difference from average	Standard error of the difference	Effect Size (d-Cohen)
ACO	2.59	3.68	0.014	6.58	2.54	0.734

ACO. asymmetry of occlusal contacts. t-Welch test (*p*<0.05).

**Table 4:** Multiple Linear Regression for ASYM and ATTIV in the model.

Variables	Squares Sum	gl	Medium Square	F	p-value	R^2^
ASYIM	3.126	1	3.126	6.35	0.015	
ATIV	0.967	1	0.967	1.96	0.168	
Residue	23.125	47	0.492			
Model Fit Index	-	-	-	-	-	0.146

ASYM. asymmetry in the electromyographic activity of the masseter and temporal muscles in the latero-lateral direction; ATIV. asymmetry in the electromyographic activity of the masseter and temporal muscles in the anteroposterior direction.

## Data Availability

The datasets used and/or analyzed during the current study are available from the corresponding author.
